# Alzheimer's Disease and Risk of Hip Fracture: A Meta-Analysis Study

**DOI:** 10.1100/2012/872173

**Published:** 2012-05-02

**Authors:** Yan Zhao, Liang Shen, Hong-Fang Ji

**Affiliations:** Shandong Provincial Research Center for Bioinformatic Engineering and Technique, Shandong University of Technology, Zibo 255049, China

## Abstract

*Background*. Alzheimer's disease (AD) is the most common cause of dementia in the elderly population. Growing evidence supports that AD patients are at high risk for hip fracture, but the issue remains questionable. The purpose of the present study is to perform a meta-analysis to explore the association between AD and risk of hip fracture. Considering that bone mineral density (BMD) acts as a strong predictor of bone fracture, we also studied the hip BMD in AD patients. *Methods*. We searched all publications in Medline, SciVerse Scopus, and Cochrane Library published up to January 2012 about the association between AD and hip fracture or hip BMD. *Results*. There are 9 studies included in the meta-analysis. The results indicate that AD patients are at higher risk for hip fracture (OR and 95% CI fixed: ES = 2.58, 95% CI = [2.03, 3.14]; dichotomous data: summary OR = 1.80, 95% CI = [1.54, 2.11]) than healthy controls. Further meta-analysis showed that AD patients have a lower hip BMD (summary SMD = −1.12, 95% CI = [−1.34, −0.90]) than healthy controls. *Conclusions*. It was found that in comparison with healthy controls AD patients are at higher risk for hip fracture and have lower hip BMD.

## 1. Introduction

 Alzheimer's disease (AD) is the most common cause of dementia among the elderly, and its prevalence is expected to rise steadily as the aging population swells [1, 2]. AD patients are at high risk for fractures, particularly of the hip fracture [3, 4]. Hip fracture is a major cause of disability among older people and is associated with more deaths and medical costs relative to all other osteoporosis-related fractures combined [5]. In recent years, there is a growing evidence supporting that patients affected by AD have high incidence of hip fracture [6–8]. We aim to perform a meta-analysis to provide a comprehensive conclusion on the association between AD and risk of hip fracture. Since bone mineral density (BMD) acts as a strong predictor of bone fractures [9], hip BMD levels in AD patients were also investigated to verify the risk of hip fracture in AD patients.

## 2. Methods

### 2.1. Information Retrieval

**Figure 1:**
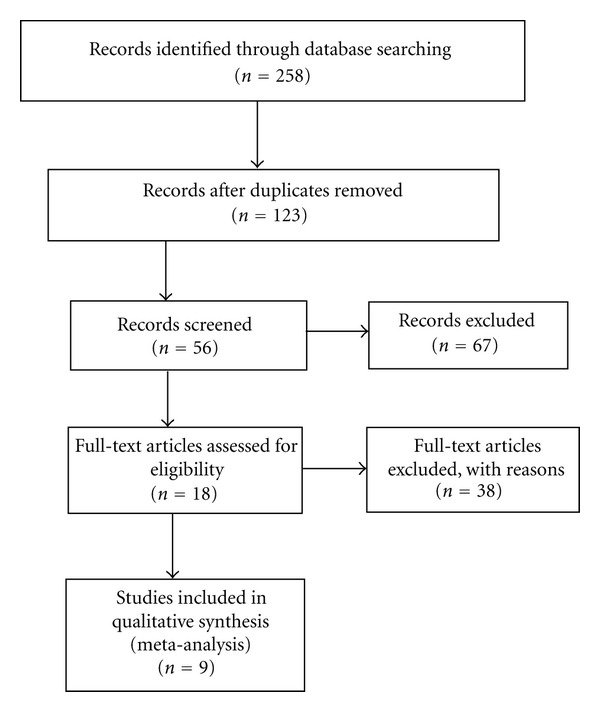
Selection of studies for inclusion in the meta-analysis.

 With the following terms: “(Alzheimer's disease and hip fracture) or (Alzheimer's disease and bone mineral density)”, a literature search was conducted in the Medline, Cochrane Library, and SciVerse Scopus databases up to January 2012. The conference proceedings and reference lists from retrieved articles were also reviewed in search of other relevant reports. We used two sets of parameters to analyze the association between AD and risk of hip fracture. The first set of parameters include the odds ratio (OR) and 95% confidence intervals (CI) fixed, and the second set of parameters include the numbers of AD patients, healthy controls, and fracture patients. As to BMD, we needed the numbers of AD patients and healthy controls, the BMD mean, and standard deviation values for both patient and healthy control groups. BMD (mm Al) was measured at the right second metacarpal using a computer-linked X-ray densitometer (CXD) (Teijin, Tokyo, Japan) [10]. In total, 9 studies were finally included in the present meta-analysis (Figure 1), among which, 3 studies (OR and 95% CI fixed) [11–13] and 4 studies (dichotomous data) [11, 12, 14, 15] reported risk of hip fracture in AD patients, and 4 studies [16–19] studied the hip BMD in AD patients.

### 2.2. Statistical Analysis

 All analyses were performed using the Stata 12.0 statistical software. We used the parameters from each study about 95% CI, OR, the standardized mean difference (SMD), and the effect size (ES). The heterogeneity was evaluated via the *I*-square test and Chi-square. If the *P* value was greater than 0.10 and the *I^2^* value was less than 50%, the study was considered not to report significant differences.

## 3. Results

### 3.1. Association between AD and Risk of Hip Fracture

**Figure 2:**
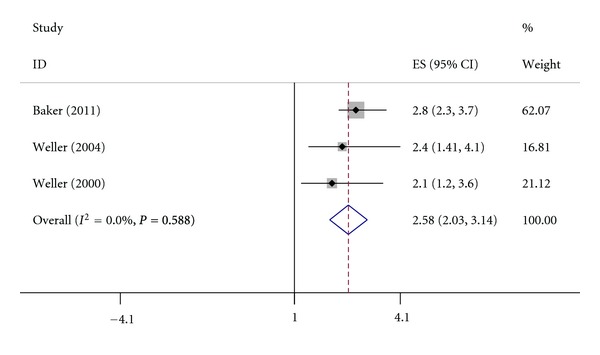
Pooled estimate of ES and 95% CI of AD and hip fracture (used OR and 95% CI fixed). ES is represented by squares, whose sizes are proportional to the sample size of the relative study. The whiskers represent the 95% CI. The diamond represents the pooled estimate based on the random effects model, with the centre representing the point estimate and the width the associated 95% CI.

**Figure 3:**
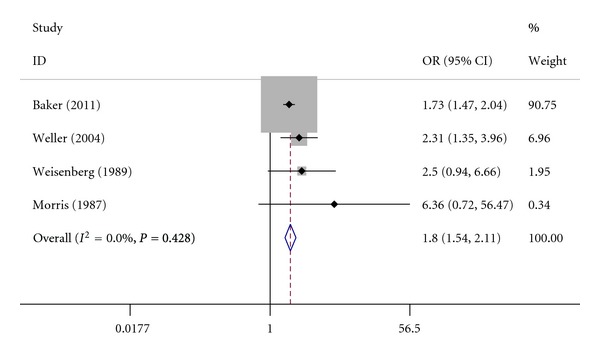
Pooled estimate of OR and 95% CI of AD and hip fracture (used dichotomous data). OR is represented by squares, whose sizes are proportional to the sample size of the relative study. The whiskers represent the 95% CI. The diamond represents the pooled estimate based on the random effects model, with the centre representing the point estimate and the width the associated 95% CI.

 There are 3 studies showing OR and 95% CI fixed and 4 studies showing dichotomous data which met the eligibility criteria.  Table 1 shows the first author, year of publication, OR, 95% CI, patient weight, ES, and 95% CI of each study.  Table 2 shows the first author, year of publication, number of AD cases and controls, number of hip fracture patients, patient weight, OR, and 95% CI of each study. The country, percentage of women patients, and average patient age of each study were also shown in Table 3. The results of the meta-analysis are shown in Figures 2 and 3, which indicate that AD patients are at higher risk for hip fracture (OR and 95% CI fixed: ES = 2.58, 95% CI = [2.03, 3.14]; dichotomous data: summary OR = 1.80, 95% CI = [1.54, 2.11]) and heterogeneity is not found among these studies (OR and 95% CI fixed: *P* = 0.588, *I^2^* = 0.0%; dichotomous data: *P* = 0.428, *I^2^* = 0.0%).

### 3.2. Association between AD and Hip BMD

**Figure 4:**
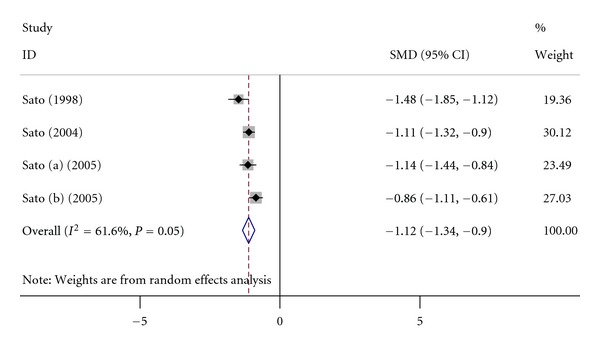
Pooled estimate of SMD and 95% CI of AD and hip BMD (mmAl). SMD is represented by squares, whose sizes are proportional to the sample size of the relative study. The whiskers represent the 95% CI. The diamond represents the pooled estimate based on the random effects model, with the centre representing the point estimate and the width the associated 95% CI.

 There are 4 studies (including 551 AD patients and 540 controls) reporting the hip BMD level in AD which met the eligibility criteria. Summary of these studies is given in Table 4. The meta-analysis results in Figure 4 indicate that AD patients have a lower hip BMD than healthy controls (summary SMD = −1.12, 95% CI = [−1.34, −0.90]).  

## 4. Discussion

 As a progressive neurodegenerative disease, the symptoms of AD include progressive loss of memory and cognitive function and apraxia [20]. Hip fracture is associated with considerable disability and loss of independence [21–23]. Recent accumulating studies indicated that both hip fracture and AD patients exhibit many similar conditions such as lower weight, lower vitamin D levels, lower gastrointestinal absorption of calcium, and higher parathyroid hormone (PTH) levels [24–28]. Some studies proposed that AD patients are at high risk for hip fracture [6–8]. This meta-analysis aims to provide a comprehensive evaluation on the association between AD and risk of hip fracture based on the available published references. The results (OR and 95% CI fixed: ES = 2.58, 95% CI = [2.03, 3.14]; dichotomous data: summary OR = 1.80, 95% CI = [1.54, 2.11]) suggest that AD patients are at higher risk for hip fracture. Moreover, it was found that AD patients have a lower hip BMD, a predictor of fracture, than healthy controls (summary SMD = −1.12, 95% CI = [−1.34, −0.90]).

 Multiple factors may help to understand the association between AD and risk of hip fracture. It has been widely reported that in comparison with healthy controls AD patients have lower levels of 25(OH)D and calcium [29, 30]. Vitamin D status is an important factor of skeletal integrity, and inadequate serum 25(OH)D level is associated with muscle weakness and increased incidences of falls and fractures [31]. Lower levels of vitamin D and calcium can also induce compensatory hyperparathyroidism, which may further contribute to a reduction in BMD [32]. Thus, it can be inferred that vitamin D and calcium deficiency may be an important factor. In addition, parathyroid hormone (PTH) may act as another important factor. Elevated PTH concentrations are associated with cognitive decline and may increase tissue aluminum loads which is a factor in the pathogenesis of AD [33, 34]. Meanwhile, it has been found that the high intact bone Gla protein and pyridinoline cross-linked carboxyterminal telopeptide of type I collagen with PTH induce compensatory hyperparathyroidism to increase bone turnover to raise the risk of fracture [17].

 The present meta-analysis has some limitations. First, many factors including anti-AD drugs, exposure to sunlight, and food intake which may influence the AD progression are not considered in the meta-analysis. Second, the number of studies included in the present study is relatively small. Third, we did not consider AD severity, which can vary the prevalence of hip fracture.

## 5. Conclusions

 To summarize, the meta-analysis indicates that AD patients are at high risk for hip fracture and have lower hip BMD than healthy controls. More efforts are warranted to elucidate the mechanisms underlying these associations.

## Figures and Tables

**Table 1:** Summary of the studies of the association between AD and hip fracture (OR and 95% CI fixed).

References	OR	95% CI	Weight (%)	ES	95% CI
[11]	2.80	[2.30, 3.70]	62.07	2.80	[2.30, 3.70]
[12]	2.40	[1.41, 4.10]	16.81	2.40	[1.41, 4.10]
[13]	2.10	[1.20, 3.60]	21.12	2.10	[1.20, 3.60]

**Table 2:** Summary of the studies of the association between AD and hip fracture (dichotomous data).

References	*N*		*N*		Weight (%)	OR	95% CI
	Fracture	AD	Fracture	Control			
[11]	391	10052	226	10052	90.75	1.73	[1.47, 2.04]
[12]	31	528	25	985	6.96	2.31	[1.35, 3.96]
[14]	10	20	12	60	1.95	2.50	[0.94, 6.66]
[15]	5	44	1	56	0.34	6.36	[0.72, 56.47]

**Table 3:** Country, gender, and mean age of the references included in the meta-analysis of AD and hip fracture.

References	Country	Gender (% female)		Mean age (year)	
		AD	Control	AD	Control
[11]	UK	65.5	—	79 ± 7.9	—
[12]	Canada	37	—	≥65	≥65
[13]	Canada	—	—	≥65	≥65
[14]	America	100	100	60	60
[15]	America	52.3	51.8	71.5 ± 5.3	71.4 ± 4.5

**Table 4:** Summary of the studies of the association between AD and hip BMD.

References	*N*		BMD (mmAl)		Weight (%)	SMD	95% CI
	AD	Control	AD	Control			
[16]	46	140	1.738 ± 0.303	2.047 ± 0.167	19.36	−1.48	[−1.85, −1.12]
[17]	205	200	2.124 ± 0.405	2.550 ± 0.360	30.12	−1.11	[−1.32, −0.90]
[18]	100	100	1.669 ± 0.324	2.000 ± 0.250	23.49	−1.14	[−1.44, −0.84]
[19]	200	100	1.885 ± 0.305	2.130 ± 0.240	27.03	−0.86	[−1.11, −0.61]
